# Association between the composite nutritional index TCBI and ISR

**DOI:** 10.3389/fnut.2026.1773370

**Published:** 2026-03-25

**Authors:** Gaoying Dai, Qi Dong, Zhaobin Sun, Nanhu Quan, Qian Tong

**Affiliations:** Department of Cardiovascular Center, The First Hospital of Jilin University, Changchun, China

**Keywords:** cardiovascular disease, cross-sectional study, ISR, nutritional index, TCBI

## Abstract

**Background:**

In-stent restenosis (ISR) is a frequent and clinically significant complication after percutaneous coronary intervention (PCI) and substantially affects long-term patient outcomes. Its underlying mechanisms are multifactorial and remain incompletely understood. The triglyceride-total cholesterol-body weight index (TCBI) has recently emerged as a novel marker of nutritional and metabolic status. However, its association with the risk of ISR has not been fully elucidated. This study aimed to investigate the value of the TCBI for predicting ISR in patients who underwent PCI.

**Methods:**

From January 2022 to January 2024, a total of 454 patients who underwent PCI were enrolled and classified into a stent-narrowing group or a stent-nonnarrowing group according to follow-up coronary angiography (CAG) findings. Baseline clinical and laboratory variables were collected, and the TCBI, atherogenic index of plasma (AIP), atherosclerosis coefficient (AC), residual cholesterol (RC) and triglyceride-glucose (TyG) level were calculated using standard formulas. Univariate and multivariate logistic regression analyses were conducted to identify factors associated with ISR. Receiver operating characteristic (ROC) curves were generated, and the area under the curve (AUC) was used to compare the predictive performance of the nutritional and metabolic indices in the overall population. Restricted cubic spline modeling and segmented regression were further applied to characterize the dose–response relationship between TCBI and ISR. Mediation analysis was performed to explore potential metabolic and inflammatory mediators. Subgroup analyses were performed to assess the robustness and consistency of the association between the TCBI and ISR across clinically relevant strata.

**Results:**

ROC analysis demonstrated that the TCBI had the highest predictive performance for ISR (AUC = 0.718, 95% CI: 0.665–0.771), outperforming the AIP, AC, RC and TyG. Elevated TCBI was independently associated with an increased risk of ISR according to both unadjusted and multivariable-adjusted logistic regression models. Restricted cubic spline and piecewise regression confirmed a nonlinear association between TCBI and ISR risk. Mediation analysis indicated that the relationship between the TCBI and ISR was partially mediated by metabolic and inflammatory factors, particularly uric acid (UA) levels and white blood cell (WBC) count. Subgroup analyses revealed that the association between the TCBI and ISR remained robust and consistent across different clinical characteristics, with no significant effect modification observed.

**Conclusion:**

AC, RC, AIP, TyG and TCBI were associated with elevated ISR risk, with TCBI having the strongest predictive value. These findings highlight TCBI as a promising metabolic–nutritional marker for ISR risk stratification and emphasize the need for further refinement of the TCBI index to enhance its utility in cardiovascular risk assessment.

## Introduction

In-stent restenosis (ISR) is defined as a ≥50% reduction in luminal diameter within a previously implanted stent or within 5 mm of its edges (1). The diagnosis of ISR was confirmed by quantitative coronary angiography (CAG) combined with visual estimation by two experienced interventional cardiologists, they were blinded to the clinical and laboratory data. Discrepancies were resolved by consensus. ISR remains among the most frequent adverse events following percutaneous coronary intervention (PCI), it has a substantial effect on long-term prognosis and quality of life. Although the advent of drug-eluting stents has significantly reduced the incidence of ISR (2), it continues to be a major cause of interventional failure and repeat revascularization in patients with coronary artery disease. The pathogenesis of ISR is multifactorial and involves complex interactions among excessive vascular smooth muscle cell proliferation, inflammatory activation, neointimal remodeling, and metabolic disturbances (3–5). However, the contribution of nutritional and metabolic status to ISR risk has received limited attention, and simple, quantifiable, and clinically applicable composite indicators are still lacking.

Several lipid-related nutritional indices, such as the atherogenic coefficient (AC) (6, 7), atherogenic index of plasma (AIP) (8, 9), remnant cholesterol (RC) (10, 11), and triglyceride-glucose (TyG), have been utilized to evaluate cardiovascular risk. These markers incorporate various combinations of lipid components, including high-density lipoprotein cholesterol (HDL-C), low-density lipoprotein cholesterol (LDL-C), total cholesterol (TC), and triglycerides (TG). The triglyceride-total cholesterol-body weight index (TCBI) is a recently introduced integrated metabolic-nutritional marker calculated from serum TG levels, TC levels, and body mass index (BMI) (12). The TCBI reflects both lipid metabolic status and nutritional condition, enabling the assessment of malnutrition while also capturing features of metabolic overload. Previous studies have linked TCBI to multiple clinical conditions, including cardiovascular disease (13, 14), postmenopausal osteoporosis (15), cognitive impairment (16, 17), diabetic nephropathy (18), stroke-associated pneumonia (19), and metabolic dysfunction-associated fatty liver disease risk (20). Nevertheless, its predictive value for ISR following PCI has not been established.

Therefore, this study aimed to comprehensively compare the predictive performance of the TCBI with that of traditional lipid-derived indices—including AIP, AC, RC, and TyG—in assessing ISR risk.

## Methods

### Study population

**Figure 1:**
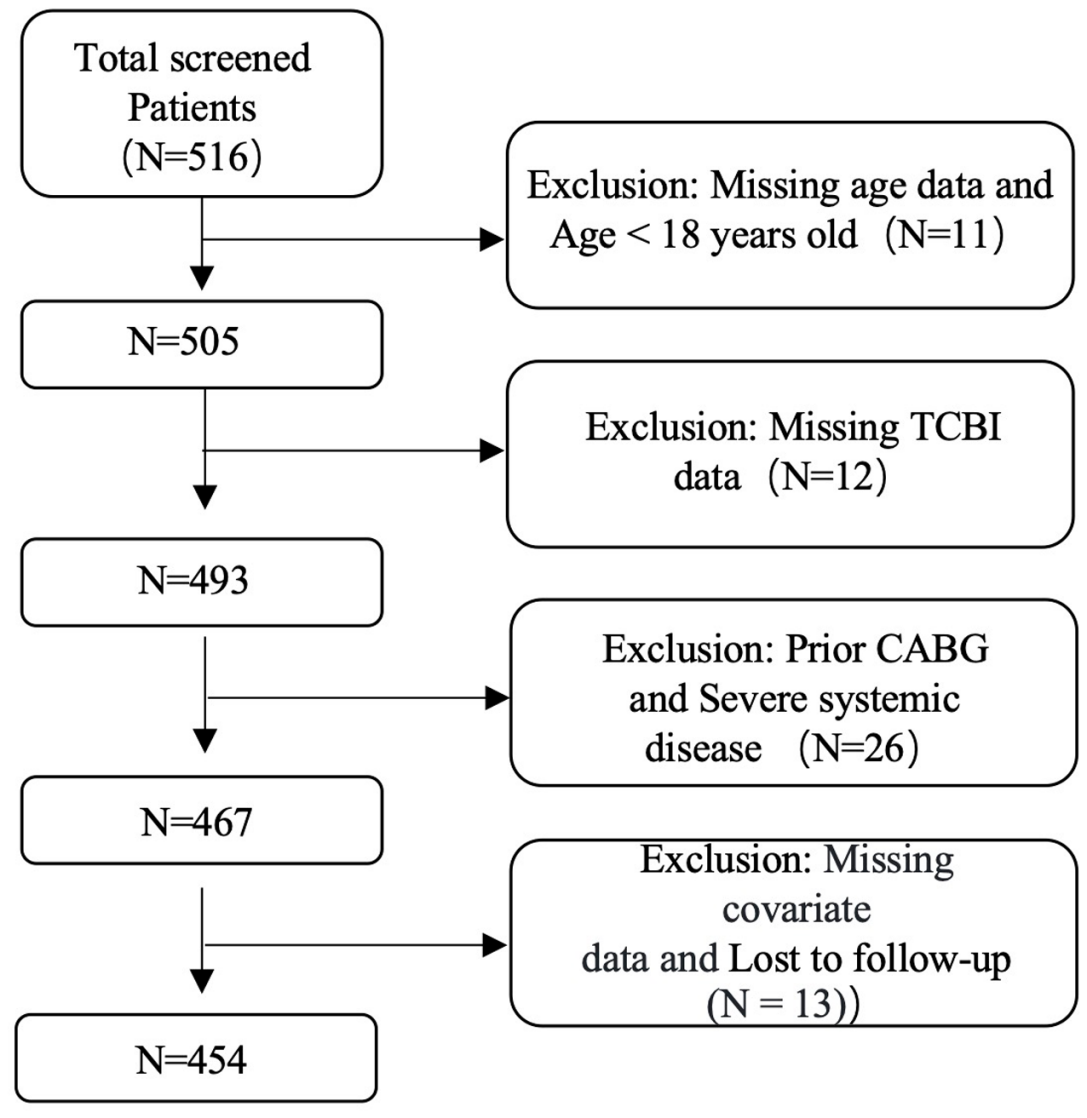
Flow chart.

Patients who were consecutively admitted to the Department of Cardiology at the First Hospital of Jilin University between January 2022 and December 2024 were screened for eligibility. Individuals were included if they were diagnosed with coronary atherosclerotic heart disease by CAG, underwent PCI with drug-eluting stent implantation, and received dual antiplatelet therapy with aspirin and clopidogrel after the procedure. All enrolled participants underwent repeat CAG at our institution within 12–24 months after discharge as part of routine clinical follow-up. A total of 454 eligible participants were included in the analysis (Figure 1).

### Inclusion criteria

Eligibility for enrollment required that patients meet all of the following criteria:

Age >18 years.
Diagnosis of coronary artery disease confirmed by CAG and treatment with at least one drug-eluting stent.
Postoperative dual antiplatelet therapy with aspirin and clopidogrel.
Underwent follow-up CAG at 12–24 months after discharge in our department.
Availability of complete clinical and laboratory data.

### Exclusion criteria

The exclusion criteria included any of the following conditions:

History of coronary artery bypass grafting (CABG).
Coexisting cardiomyopathy or valvular heart disease.
Presence of malignant tumors.
Severe systemic illness or autoimmune disease.
Incomplete medical records or missing key clinical information.
Calculation of metabolic and nutritional indices.

The following lipid-related nutritional indices were calculated using standard formulas:


$$\text{TCBI}=\frac{\mathrm{TG}\;(\mathrm{mg}/\mathrm{dL})\times \mathrm{TC}\;(\mathrm{mg}/\mathrm{dL})\times \mathrm{BW}\;(\mathrm{kg})}{1,000}$$

RC=TC(mg/dL)−HDL−C(mg/dL)−LDL(mg/dL)

AC=(TC(mg/dL)−HDL−C(mg/dL))/HDL−C(mg/dL)

AIP=log[TG(mmol/L)/HDL−C(mmol/L)]

TyG=ln[TG(mg/dL)×FBG(mg/dL)/2]

### Covariates

Demographic and health-related variables were included as covariates in the analysis. The demographic factors included age, sex, and other baseline characteristics. Health-related factors included BMI, diabetes status, hypertension status, and current smoking and drinking status. BMI was calculated as weight divided by height squared (kg/m^2^). Laboratory parameters included lymphocyte count, white blood cell (WBC) count, serum creatinine (Cr) level, serum uric acid (UA) level, TC level, LDL-C level, HDL-C level, and TG level.

Hypertension was defined as a self-reported history of hypertension or blood pressure measurements meeting diagnostic criteria (diastolic blood pressure ≥90 mmHg or systolic blood pressure ≥140 mmHg). Diabetes was defined as a self-reported physician diagnosis or a fasting plasma glucose (FPG) concentration ≥126 mg/dL or a glycated hemoglobin (HbA1c) concentration ≥6.5%.

Demographic information was retrieved from the hospital electronic medical records system, laboratory data from the Laboratory Information System (LIS), and imaging data from the Siemens QCA System. All patients underwent standardized interviews and medical history verification at admission, with information confirmed by the patient or family members. Laboratory tests—including liver and renal function tests, fasting glucose levels, lipid profiles, HbA1c levels, and transthoracic echocardiography—were performed at our hospital within 2 days prior to CAG. Postdischarge medication adherence was assessed through structured telephone follow-up.

### Statistical analysis

The normality of continuous variables was assessed using the Kolmogorov–Smirnov test. Normally distributed data were compared with the *t*-test (mean ± SD), while nonnormally distributed data were compared using the Mann–Whitney *U* test (median, interquartile range). Categorical variables were analyzed with the chi-square test and are expressed as frequencies (%). Logistic regression models were constructed as follows: Model 1, unadjusted; Model 2, adjusted for age and sex; Model 3, further adjusted for BMI, smoking status, alcohol use status, hypertension status, diabetes status, and myocardial infarction status. Restricted cubic splines (RCS) were performed using the “rms” R package to evaluate potential nonlinear relationships between nutritional indices and ISR. Receiver operating characteristic (ROC) analysis was performed with the “pROC” package, and differences in the area under the curve (AUC) were tested by the DeLong method. Two-sided *p* < 0.05 indicated statistical significance. Analyses were conducted in R (version 4.5).

## Results

### Baseline characteristics

A total of 454 patients who underwent PCI were included, of whom 194 (42.7%) developed ISR and 260 (57.3%) did not. Compared with non-ISR patients, ISR patients were older [median 64.0 (57.0–71.0) vs. 62.0 (55.0–70.0) years], had a higher BMI (25.07 vs. 24.22 kg/m^2^), and had a greater prevalence of diabetes (44% vs. 33%) and smoking (42% vs. 30%). Biochemical analysis revealed that ISR patients had higher levels of TC, TG, and LDL-C, whereas HDL-C did not significantly differ. Atherosclerosis-related indices, including AIP, AC, AI, RC, and TCBI, were significantly elevated in the ISR group. ISR patients also had higher UA levels, Cr levels, fasting glucose levels, HbA1c levels, and WBC counts and lower left ventricular ejection fraction (LVEF). CAG demonstrated that ISR patients more multivessel disease (MVD, 77% vs. 51%), calcified lesions (24% vs. 6.9%), and chronic total occlusions (CTO, 43% vs. 20%). All *p*-values were <0.05 (Table 1).

**Table 1:** Baseline characteristics.

Characteristic	Overall *N* = 454^1^	Non-ISR *N* = 260^1^	ISR *N* = 194^1^	*p*-value^2^
Gender				0.030
Female	159 (35%)	102 (39%)	57 (29%)	
Male	295 (65%)	158 (61%)	137 (71%)	
Age (years)	63 (56, 70)	62 (55, 70)	64 (57, 71)	0.100
Height (m)	1.70 (1.60, 1.73)	1.68 (1.60, 1.72)	1.70 (1.62, 1.73)	0.100
Weight (kg)	70 (62, 77)	70 (60, 75)	71 (65, 80)	0.002
Smoke				0.005
No	295 (65%)	183 (70%)	112 (58%)	
Yes	159 (35%)	77 (30%)	82 (42%)	
Drink				0.700
No	390 (86%)	225 (87%)	165 (85%)	
Yes	64 (14%)	35 (13%)	29 (15%)	
Hypertension				0.500
No	193 (43%)	107 (41%)	86 (44%)	
Yes	261 (57%)	153 (59%)	108 (56%)	
Diabetes				0.014
No	282 (62%)	174 (67%)	108 (56%)	
Yes	172 (38%)	86 (33%)	86 (44%)	
Alb (g/L)	39.0 (36.6, 41.5)	38.8 (36.6, 41.5)	39.1 (36.4, 41.5)	0.800
UA, μmol/L	339 (272, 400)	330 (268, 385)	364 (293, 420)	0.005
Cr, μmol/L	71 (59, 86)	69 (57, 82)	74 (63, 90)	0.009
FBG, mmol/L	5.82 (5.11, 7.43)	5.67 (5.06, 6.92)	6.10 (5.25, 8.59)	0.003
HbA1c, %	6.30 (5.80, 7.20)	6.20 (5.70, 6.90)	6.40 (5.80, 7.90)	0.005
LVEF, %	61 (55, 64)	62 (57, 64)	59 (52, 63)	0.005
BMI, kg/m^2^	24.49 (22.99, 26.57)	24.22 (22.63, 26.20)	25.07 (23.66, 27.04)	0.002
WBC (10^9^/L)	7.41 (6.14, 9.11)	7.37 (6.10, 8.76)	7.63 (6.29, 9.60)	0.100
LY (10^9^/L)	1.88 (1.46, 2.44)	1.92 (1.52, 2.51)	1.79 (1.41, 2.35)	0.065
MVD				<0.001
No	171 (38%)	126 (49%)	45 (23%)	
Yes	282 (62%)	133 (51%)	149 (77%)	
Bifurcation lesion				0.055
No	395 (87%)	233 (90%)	162 (84%)	
Yes	59 (13%)	27 (10%)	32 (16%)	
Calcified lesion				<0.001
No	389 (86%)	242 (93%)	147 (76%)	
Yes	65 (14%)	18 (6.9%)	47 (24%)	
CTO				<0.001
No	319 (70%)	209 (80%)	110 (57%)	
Yes	135 (30%)	51 (20%)	84 (43%)	
MI				0.007
No	218 (48%)	139 (53%)	79 (41%)	
Yes	236 (52%)	121 (47%)	115 (59%)	
AIP	0.55 (0.39, 0.73)	0.51 (0.34, 0.69)	0.62 (0.46, 0.78)	<0.001
AC	2.81 (2.27, 3.49)	2.58 (2.11, 3.15)	3.08 (2.66, 3.88)	<0.001
AI	1.27 (0.90, 1.68)	1.17 (0.78, 1.58)	1.43 (1.05, 1.80)	<0.001
RC (mg/dL)	18 (12, 25)	15 (10, 22)	21 (15, 29)	<0.001
TCBI	1,383 (912, 2,296)	1,156 (797, 1,697)	1,915 (1,139, 3,088)	<0.001
TC, mg/dL	147 (128, 169)	137 (122, 159)	159 (141, 187)	<0.001
TG, mg/dL	138 (97, 198)	124 (93, 173)	163 (111, 231)	<0.001
HDL, mg/dL	38 (33, 44)	38 (33, 44)	39 (34, 44)	0.600
LDL, mg/dL	90 (73, 106)	81 (68, 99)	99 (84, 119)	<0.001

BMI, body mass index; HDL, high-density lipoprotein; LDL, low-density lipoprotein; UA, uric acid; HbA1c, glycosylated hemoglobin; TG, triglyceride; FBG, fasting blood glucose; TCBI, triglyceride-total cholesterol-body weight index; AIP, atherogenic index of plasma; AC, atherogenic coefficient; RC, remnant cholesterol; MI, myocardial infarction; CTO, chronic total occlusion; MVD, multi-vessel disease; LVEF, left ventricular ejection fraction; Alb, albumin; WBC, white blood cell; LY, lymphocyte count; Cr, creatinine. ^1^n (%); Median (Q1, Q3); ^2^Pearson’s Chi-squared test; Wilcoxon rank sum test.

### Comparative analysis of the predictive value for ISR among the four nutritional indices

**Figure 2:**
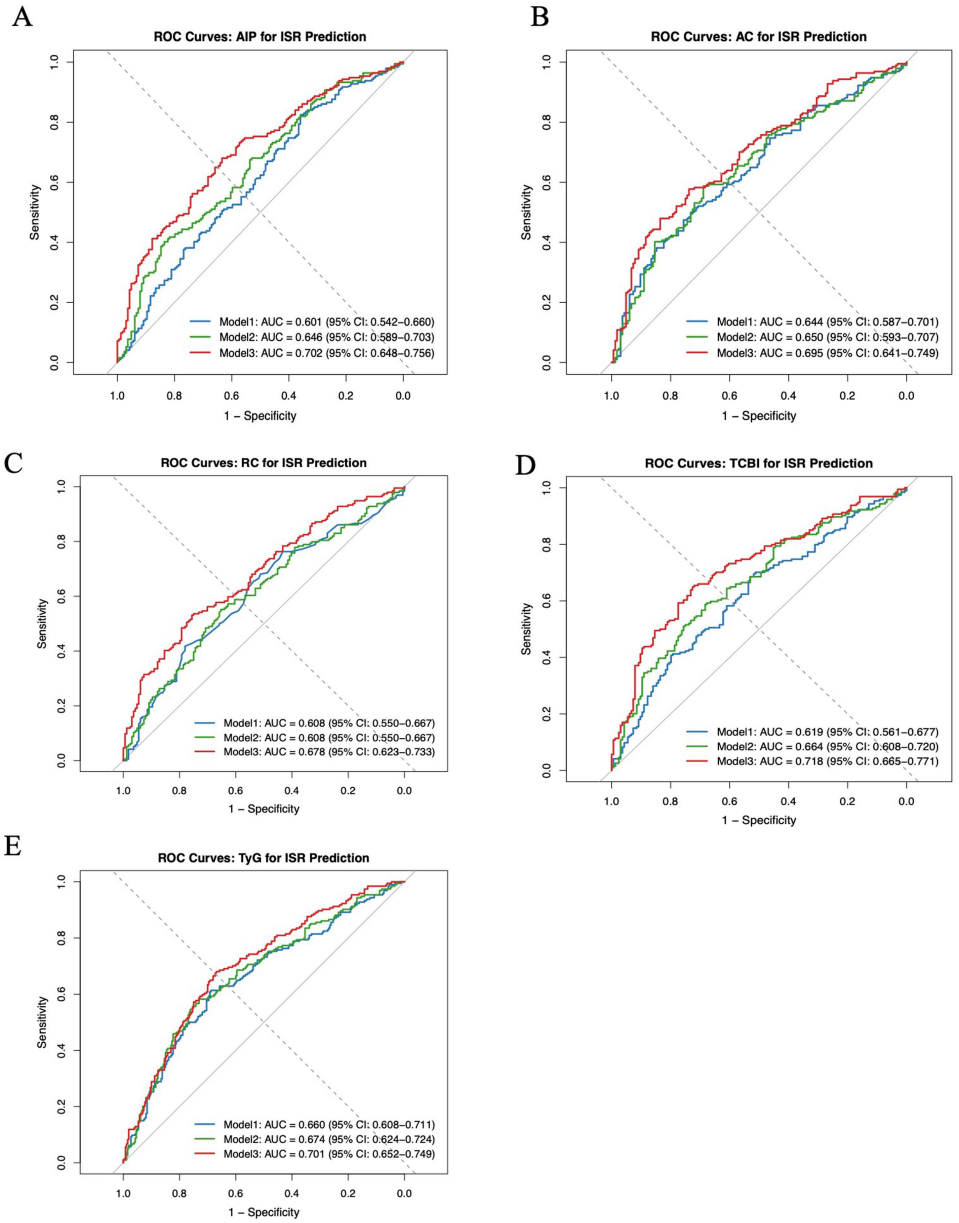
ROC curves for predicting ISR. **(A)** ROC curve for predicting ISR using AIP. **(B)** ROC curve for predicting ISR using AC. **(C)** ROC curve for predicting ISR using RC. **(D)** ROC curve for predicting ISR using TCBI. **(E)** ROC curve for predicting ISR using TyG. ROC, receiver operating characteristic; AUC, area under the curve; TCBI, triglyceride-total cholesterol-body weight index; AIP, atherogenic index of plasma; AC, atherogenic coefficient; RC, remnant cholesterol; TyG, triglyceride-glucose index.

ROC curve analysis was performed to compare the predictive value of the AIP, AC, TCBI, TyG, and RC for ISR (Figure 2). Three models were constructed: Model 1, unadjusted; Model 2, adjusted for age and sex; and Model 3, further adjusted for BMI, smoking status, alcohol use status, hypertension status, diabetes status, and prior myocardial infarction status. In Model 3, TCBI demonstrated the highest predictive performance, with an AUC of 0.718 (95% CI: 0.665–0.771), outperforming AC (AUC 0.695, 95% CI: 0.641–0.749), AIP (AUC 0.702, 95% CI: 0.648–0.756), and TyG (AUC 0.701, 95% CI: 0.652–0.749), whereas RC had the lowest AUC (0.678, 95% CI: 0.623–0.733). Consistent patterns were observed in Models 1 and 2.

### Associations between TCBI and the risk of ISR

Table 2 summarizes the associations between TCBI and ISR risk across the three logistic regression models. When treated as a continuous variable, log-transformed TCBI was strongly associated with ISR. In the unadjusted model (Model 1), each unit increase in log (TCBI) nearly tripled the odds of ISR (OR = 2.98, 95% CI: 2.17–4.15, *p* < 0.001). This association remained robust after adjusting for age and sex in Model 2 (OR = 2.94; 95% CI: 2.13–4.13; *p* < 0.001) and persisted in Model 3, which included BMI, smoking status, alcohol use, hypertension status, diabetes status, and prior myocardial infarction status (OR = 2.71; 95% CI: 1.94–3.84; *p* < 0.001). When TCBI was analyzed by quartiles, a clear dose–response relationship was observed. Compared with the lowest quartile (Q1), the ISR risk progressively increased across the higher quartiles. In the fully adjusted model, participants in Q3 had a 1.85-fold higher risk (OR = 1.85, 95% CI: 1.02–3.40; *p* = 0.045), whereas those in Q4 exhibited a more than fivefold increase in ISR risk (OR = 5.22, 95% CI: 2.84–9.82; *p* < 0.001).

**Table 2:** Logistics regression analysis of TCBI with ISR.

Group		Model 1		Model 2		Model 3	
	Characteristic	OR (95% CI)	*p*-value	OR (95% CI)	*p*-value	OR (95% CI)	*p*-value
TCBI Continuous	log_TCBI	2.98 (2.17, 4.15)	<0.001	2.94 (2.13, 4.13)	<0.001	2.71 (1.94, 3.84)	<0.001
TCBI Categorical	TCBI						
	Q1	—		—		—	
	Q2	1.82 (1.03, 3.24)	0.041	1.74 (0.98, 3.13)	0.059	1.62 (0.89, 2.95)	0.120
	Q3	2.19 (1.25, 3.89)	0.007	2.14 (1.21, 3.84)	0.010	1.85 (1.02, 3.40)	0.045
	Q4	6.39 (3.62, 11.6)	<0.001	6.10 (3.39, 11.2)	<0.001	5.22 (2.84, 9.82)	<0.001

Model 1: unadjusted. Model 2: adjusted for age, sex. Model 3: adjusted for age, sex, BMI, smoking, alcohol use, hypertension, diabetes, and myocardial infarction.

95% CI, 95% confidence interval; OR, odds ratio; TCBI, triglyceride-total cholesterol-body weight index; BMI, body mass index.

### Assessment of nonlinear relationships and piecewise regression

**Figure 3:**
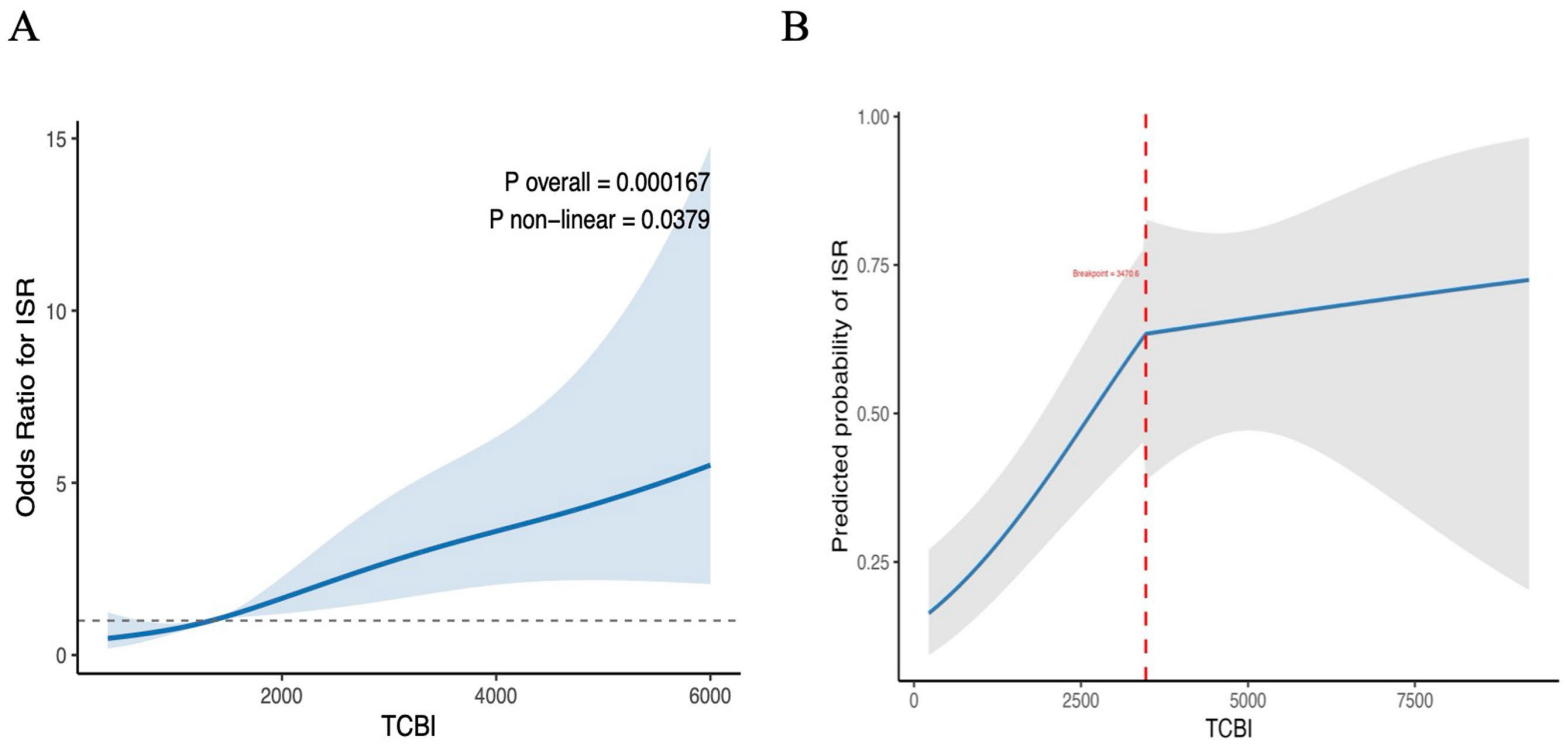
Restricted spline curve for the association between TCBI and ISR. The blue line in panel **(A)** represents the OR, with the blue transparent area indicating the 95% confidence interval. The gray line in panel **(B)** represents predictive capability, with the gray transparent area indicating the 95% confidence interval. OR (95% CI) values were adjusted based on Model 3. **(A)** Association between TCBI and ISR. **(B)** Segmented regression fit between TCBI and ISR. TCBI, triglyceride-total cholesterol-body weight index; ISR, in-stent restenosis.

To examine potential nonlinear associations between nutrition-related indices and ISR, restricted cubic spline (RCS) analysis was performed using Model 3 (Figure 3). The RCS revealed a significant nonlinear relationship between the TCBI and ISR risk (*p* for nonlinearity <0.05). Piecewise regression revealed an inflection point at TCBI ≈3470.6 (SE = 948.7), indicating a marked change in slope. Below this threshold, higher TCBI was positively associated with ISR risk (*β* = 0.000672, *p* < 0.001). Sensitivity analysis using bootstrap resampling validated the stability of the TCBI threshold (3470.6). The bootstrapped thresholds had a mean of 2459.0 (median = 2618.1) and a 95% CI of (515.4, 3951.5), indicating good stability. Beyond this point, the slope attenuated substantially (*β* = −0.000599), reflecting a plateau effect and suggesting saturation of the metabolic load, whereby the impact of the TCBI on the ISR diminished at levels of approximately 3,500. In the covariate-adjusted model, age (*p* = 0.0299), smoking status (*p* = 0.0164), diabetes status (*p* = 0.0113), and prior myocardial infarction status (*p* = 0.0436) remained independent predictors of ISR, whereas sex, hypertension status, and alcohol consumption were not statistically significant (*p* > 0.05). Compared with a linear specification (AIC = 567.5), incorporating a piecewise structure improved the model fit, supporting a threshold-dependent association between the TCBI and ISR.

### Subgroup analysis

**Figure 4:**
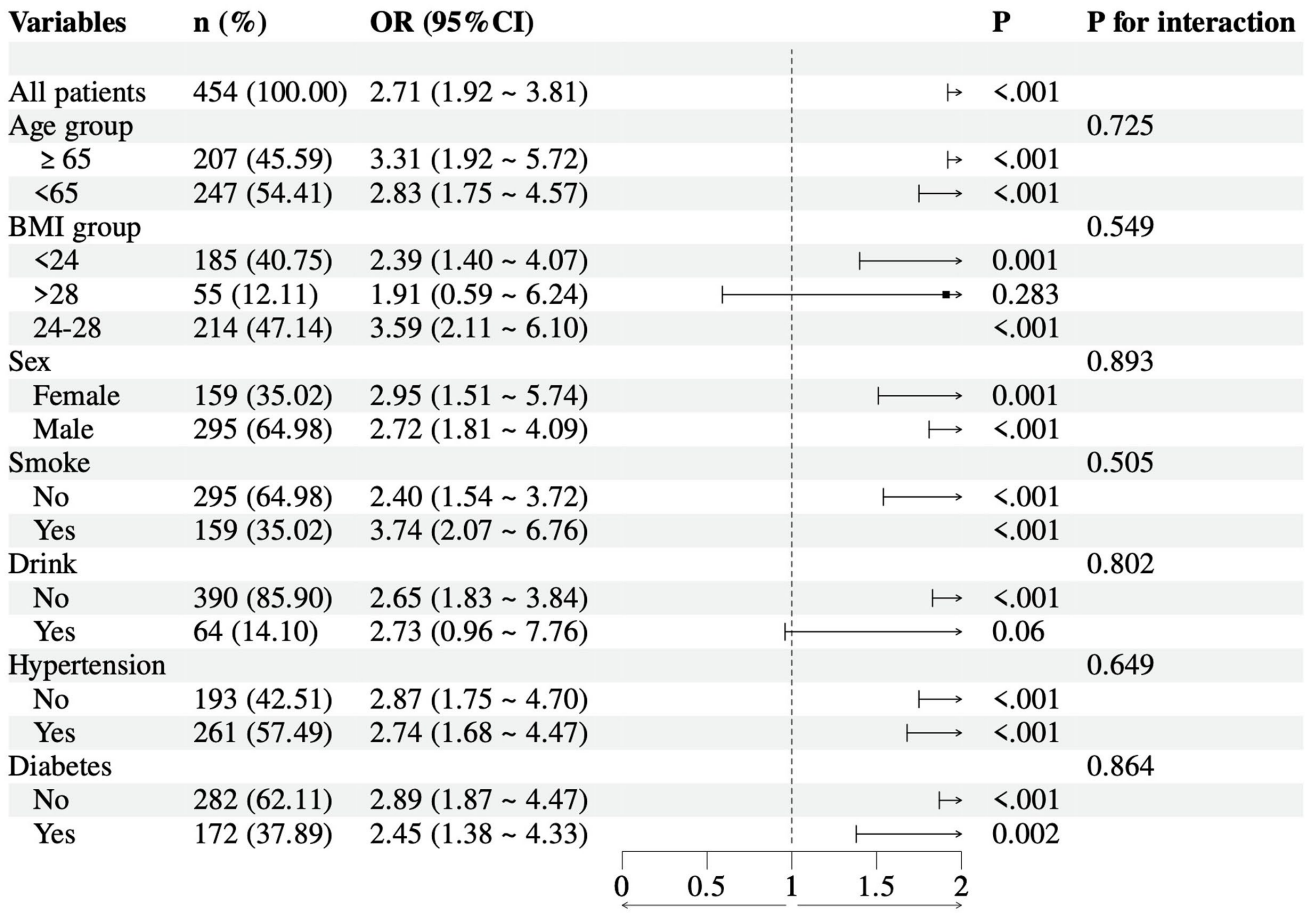
Subgroup analysis of association of TCBI with ISR.

To evaluate the consistency of the association between the TCBI and ISR across different patient populations, subgroup analyses were conducted on the basis of key demographic and clinical characteristics (Figure 4). A positive association between TCBI and ISR risk was observed in the overall population (OR = 2.71, 95% CI: 1.92–3.81, *p* < 0.001) and across all the examined subgroups. Interaction tests indicated no significant effect modification by any factor (all *p*-values for interactions >0.05). The association remained significant across age (≥65 years: OR = 3.31, *p* < 0.001; <65 years: OR = 2.83, *p* < 0.001; interaction *p* = 0.725), sex (male: OR = 2.72, *p* < 0.001; female: OR = 2.95, *p* = 0.001; interaction *p* = 0.893), and BMI categories (BMI <24: OR = 2.39, *p* = 0.001; BMI 24–28: OR = 3.59, *p* < 0.001; BMI ≥28: OR = 1.91, *p* = 0.283; interaction *p* = 0.549). Consistent associations were also observed in subgroups stratified by smoking status (nonsmokers: OR = 2.40; *p* < 0.001; smokers: OR = 3.74; *p* < 0.001; interaction *p* = 0.505) and comorbidities, including hypertension (yes: OR = 2.74; *p* < 0.001; no: OR = 2.87; *p* < 0.001; interaction *p* = 0.649) and diabetes (yes: OR = 2.45; *p* = 0.002; no: OR = 2.89; *p* < 0.001; interaction *p* = 0.864).

### Mediation analysis

Logistic regression analysis revealed a significant association between the TCBI and ISR risk. Considering the complex pathogenesis of ISR, we hypothesized that TCBI may exert its effects through intermediate biological pathways, including fasting blood glucose (FBG), LVEF, WBC, UA, albumin, Cr, and lymphocyte count. To formally test this hypothesis and quantify potential indirect effects, a mediation analysis was performed. Table 3 summarizes the associations between TCBI and candidate mediators (Path a) and between mediators and ISR (Path b). After adjustment for age, sex, BMI, smoking status, alcohol consumption, hypertension status, diabetes status, and prior myocardial infarction status, a mediation analysis revealed that UA level and WBC count significantly mediated the relationship between the TCBI score and ISR. Specifically, TCBI was positively associated with UA (*β* = 0.0056; *p* = 0.0199), which in turn was positively associated with ISR (*β* = 0.0028; *p* = 0.0292), indicating a significant indirect effect. Similarly, the TCBI correlated with the WBC count (*β* = 0.00029; *p* < 0.001), and an elevated WBC count was independently associated with increased ISR risk (*β* = 0.123; *p* = 0.0229). In contrast, other candidate mediators—including albumin, Cr, FBG, LVEF, and lymphocyte count—did not demonstrate significant indirect effects (*p* > 0.05).

**Table 3:** Summary of mediation analysis.

	Path a		Path b	
Mediator	Estimate	*p*-value	Estimate	*p*-value
Alb	4.66 × 10^−5^	0.615	0.011	0.735
Cr	3.39 × 10^−4^	0.643	0.007	0.145
FBG	1.82 × 10^−4^	<0.001	0.056	0.349
LVEF	8.70 × 10^−4^	<0.001	0.012	0.360
LY	8.33 × 10^−7^	0.967	0.249	0.100
UA	5.57 × 10^−3^	0.020	0.003	0.029
WBC	2.88 × 10^−4^	<0.001	0.123	0.023

Alb, albumin; Cr, creatinine; FBG, fasting blood glucose; LVEF, left ventricular ejection fraction; LY, lymphocyte count; UA, uric acid; WBC, white blood cell. Path a: TCBI → Mediator. Path b: Mediator → ISR.

## Discussion

In this study, 454 patients who underwent PCI were analyzed, and ISR occurrence was evaluated using four lipid-derived indices. Among these indicators, the TCBI demonstrated the strongest and most consistent association with ISR, particularly in elderly patients, individuals with diabetes, and smokers. The association remained robust across all the models, regardless of whether TCBI was analyzed as a continuous or categorical variable. Moreover, both segmented regression and RCS analyses revealed a distinct nonlinear relationship between the TCBI and ISR, indicating that increases in the TCBI are associated with elevated ISR risk up to a threshold beyond which the effect plateaus.

The superior predictive performance of the TCBI may be attributed to its ability to integrate three key metabolic components—triglycerides, total cholesterol, and body mass index. Previous studies have demonstrated that the TCBI is independently associated with multiple cardiovascular endpoints, including CVD mortality, stroke, and long-term prognosis, in patients with coronary artery disease. In a large prospective cohort (MASHAD study, *n* = 9,704), the TCBI was significantly associated with increased CVD mortality (HR = 1.078, 95% CI: 1.012–1.15) over 10 years, whereas the AIP and AC were not associated (21). In the CHARLS cohort (*n* = 8,104), the TCBI was strongly predictive of incident CVD (HR = 1.59, 95% CI: 1.31–1.92) during the 9-year follow-up, with a short-term predictive performance comparable to that of the TyG-BMI (22). In patients who underwent PCI, an elevated TCBI was related to reduced all-cause and cardiovascular mortality, supporting its role as a favorable prognostic nutritional marker (12). Notably, in a large Chinese hypertensive cohort (*n* = 13,358), higher TCBI was independently associated with lower stroke incidence, especially in adults younger than 60 years, suggesting population- and outcome-specific associations (23). Thus, emerging evidence confirms that TCBI is a biologically meaningful and clinically useful metabolic–nutritional marker.

To elucidate the potential mechanistic link between TCBI and ISR, we first investigated the central signaling cascades governing metabolic homeostasis. The mammalian target of rapamycin (mTOR) signaling pathway serves as a central sensor for nutrients and energy and plays a pivotal role in orchestrating metabolic regulation (24, 25). However, its functional role as an intermediate mechanism underlying TCBI-related ISR remains largely hypothetical. The mTOR pathway is activated by nutritional signals such as glucose and lipids, modulates lipogenesis, and maintains metabolic homeostasis. Its aberrant activation leads to hyperinsulinemia, obesity, and liver steatosis (26–28), which are highly consistent with the core characteristics of elevated TCBI. In vascular remodeling, the mTOR pathway promotes endothelial repair, abnormal proliferation and migration of vascular smooth muscle cells, and collagen synthesis (29), thereby exacerbating ISR. Given these findings, we propose a hypothetical model in which the mTOR pathway may serve as a key intermediate hub mediating the regulatory effect of TCBI on ISR. Elevated TCBI may activate the mTOR pathway, which in turn induces metabolic disturbance, amplifies inflammatory responses, and accelerates vascular remodeling. These processes collectively promote neointimal hyperplasia after stent implantation and increase the risk of ISR.

Beyond the potential mechanistic link between the TCBI and ISR, the nonlinear relationship observed between the TCBI and ISR risk may be explored through distinct biological mechanisms. First, as a marker of metabolic overload, TCBI integrates lipids, cholesterol, and weight. Below a critical threshold, adaptive vascular defenses mitigate minor metabolic abnormalities, lowering the risk of ISR. Above the threshold, excessive lipids, inflammation, and oxidative stress induce endothelial dysfunction, smooth muscle proliferation, and matrix deposition. Second, the threshold denotes a shift from homeostasis to pathology. Beyond this point, metabolic stress synergistically drives inflammation and plaque instability, accelerating neointimal hyperplasia. Notably, our data revealed a diminished effect at extremely high levels of TCBI, likely attributed to compensatory feedback inhibition or metabolic compensation (consistent with the obesity paradox). These adaptive pathways form an integrated buffering system that modulates ISR risk (30–32).

Mediation analysis provided more critical insights into the causal pathway connecting the TCBI to the ISR. Among the candidate mediators evaluated, UA and WBC exhibited statistically significant mediating effects. The TCBI was positively associated with serum UA levels, which were independently positively related to the risk of ISR. Similarly, a higher TCBI was significantly associated with elevated WBC counts, which in turn independently predicted ISR risk. This indirect pathway implies that individuals with higher TCBI may experience an increased purine metabolic burden and vascular inflammation, resulting in elevated oxidative stress and endothelial dysfunction, these mechanisms are well recognized as drivers of neointimal hyperplasia and restenosis (4, 33–36). These findings indicate that the relationship between TCBI and ISR is partly mediated through inflammatory and purine metabolism related pathways, highlighting potential biological mechanisms. Specifically, these mechanisms underlie how metabolic–nutritional burden affects vascular healing following stent implantation.

After stratification and full adjustment for covariates, the association between the TCBI and ISR remained consistent across all the examined subgroups, with no significant interaction effects. Although the association in the BMI ≥28 subgroup did not reach statistical significance, this may be attributed to the relatively small sample size and limited statistical power in this subgroup. In addition, individuals with obesity usually present with more complex metabolic disorders and comorbidities, which may also weaken the observed association, and the overall relationship between TCBI and ISR was not materially modified by BMI. This robustness suggests that the TCBI captures a shared metabolic–inflammatory burden across diverse clinical characteristics, making it a stable and reliable indicator of ISR risk.

In summary, ISR development reflects the combined influence of aging, metabolic abnormalities, systemic inflammation, and lesion complexity. Our findings reaffirm the central role of atherosclerotic and inflammatory pathways in restenosis formation and suggest that more stringent post-PCI management—including tighter control of lipid profiles, glycemic status, BMI, and inflammatory markers—may help mitigate ISR risk.

### Limitations

This study has several limitations. First, its retrospective, single-center design inherently restricts causal inference. Second, several important confounding factors, including endothelial function, medication adherence, and specific inflammatory cell subtypes, were not evaluated in the present study. In addition, other critical potential confounders, such as stent type and lesion characteristics, were not included in the adjustment model. These unmeasured factors may have potential confounding effects and should be carefully considered when the current findings are interpreted. Third, this was a single-center retrospective study, which may limit the generalizability of the findings. Only patients who underwent percutaneous coronary intervention and completed follow-up were included, while those with missing data, those who were lost to follow-up, or those with severe clinical conditions were excluded. Furthermore, variations in clinical decision-making, as well as unmeasured confounders such as lifestyle and family history, may also have influenced participant selection. Another limitation is that the number of ISR events was relatively small, which prevented us from performing a time-stratified analysis to compare early and late ISR. Future studies with larger cohorts are needed to address this issue.

## Conclusion

In conclusion, this study demonstrated a significant association between the TCBI composite index and the risk of ISR following PCI. Elevated TCBI was positively and dose-dependently associated with ISR risk and exhibited a nonlinear, plateau-like pattern. Compared with traditional lipid-derived indices such as AIP, AC, and RC, TCBI showed superior predictive performance, underscoring its potential as a comprehensive metabolic and nutritional marker in ISR risk assessment. Furthermore, our findings suggest that metabolic overload and systemic inflammation may jointly contribute to ISR development. As a simple and clinically accessible indicator, the TCBI may facilitate early identification of patients at high ISR risk. Future prospective studies are warranted to validate these findings and to determine whether improving metabolic status and attenuating systemic inflammation can effectively reduce the incidence of ISR.

## Data Availability

The raw data supporting the conclusions of this article will be made available by the authors, without undue reservation.
